# Comparative Study of 1444 nm Laser Monotherapy versus Integrated Liposuction in the Treatment of Axillary Osmidrosis

**DOI:** 10.3390/medicina60071151

**Published:** 2024-07-17

**Authors:** Jae Hoon Jeong, Chongsoo Park

**Affiliations:** 1 Department of Plastic and Reconstructive Surgery, Seoul National University College of Medicine, Seoul National University Bundang Hospital, Seongnam-si 13620, Republic of Korea; drj2h@snubh.org; 2 Department of Plastic and Reconstructive Surgery, Inje University College of Medicine, Inje University Busan Paik Hospital, Busan 47392, Republic of Korea

**Keywords:** osmidrosis, axillary osmidrosis, liposuction, laser, 1444 nm wavelength

## Abstract

*Background and Objectives:* The 1444 nm wavelength Neodymium:Yttrium–Aluminum–Garnet (Nd:YAG) laser treatment is an efficient method for treating axillary osmidrosis (AO); however, it has a relatively low treatment persistence. To address this issue, we performed integrated liposuction surgery with a laser to treat AO and compared the results with those of a group treated only with a laser. *Materials and Methods:* This study compared the outcomes of AO treatment between the two groups up to six months postoperatively. The first group of 18 patients underwent laser treatment alone, and the second group of 12 patients underwent integrated liposuction surgery in addition to laser treatment. Outcomes were assessed using the following variables: degree of malodor (DOM), sweating area, patient satisfaction, pain levels, and complications, such as burns, swelling, and contractures. *Results*: Compared to the laser-only group, the integrated liposuction group demonstrated significantly superior outcomes in terms of DOM (*p* = 0.002) and patient satisfaction (*p* = 0.006), as well as a reduction in the sweating area (*p* = 0.012). The pain rating was higher in the liposuction group, but the difference was not statistically significant (*p* = 0.054). Compared with the patients in the integrated liposuction treatment group, those in the laser treatment group exhibited a significantly higher number of burns under the axillae (*p* = 0.025). However, no significant differences were observed in the swelling or contracture between the groups. *Conclusions*: Integrated liposuction with laser therapy significantly improved treatment outcomes, including malodor, patient satisfaction, sweat test results, and decreased complication rates.

## 1. Introduction

Axillary osmidrosis (AO), commonly referred to as malodorous armpits, is a dermatological condition characterized by the emission of unpleasant odors from the underarm area. This condition primarily arises from the activity of the apocrine sweat glands, which are abundant in the axillary region [1]. These glands secrete protein-rich sweat that is broken down by resident skin bacteria, leading to the release of volatile organic compounds (VOCs), which are the main cause of unpleasant odors [2]. 

The prevalence of AO varies significantly among populations and is influenced by various factors [3]. Genetic predispositions play a crucial role in this process. Specific gene variants are associated with a higher incidence of this condition and are more common in certain populations [4]. Hormonal fluctuations associated with puberty, stress, and the menstrual cycle can exacerbate apocrine gland activity, thereby increasing odor production. Furthermore, personal hygiene practices, such as the frequency of washing and use of underarm products, can significantly affect odor severity.

Despite its nonthreatening nature to physical health, AO can severely affect an individual’s psychological and social well-being. Afflicted individuals may experience social embarrassment, reduced self-esteem, and a tendency to avoid close physical contact, potentially resulting in social isolation [5].

The current treatment modalities for AO vary in approach and effectiveness. Over-the-counter solutions, such as antiperspirants and deodorants, provide temporary relief by masking odors or inhibiting sweat production. More invasive interventions, such as botulinum toxin injections, directly target the sweat glands to decrease their secretion [6]. Surgical procedures, including excision of sweat glands, offer a longer-lasting solution but carry greater risks and have variable success rates. Each treatment option presents unique limitations and potential side effects, underscoring the need for ongoing research to develop safer, more effective, and more sustainable alternatives.

This paper compares laser monotherapy and integrated liposuction with laser treatment over a six-month period. In addition, a review of the pathophysiology, epidemiology, and clinical manifestations of AO was presented. We will examine the current treatment modalities and their limitations as well as recent advances in treatment approaches. By exploring emerging research, we aim to better understand the genetic and molecular foundations of this condition and develop targeted therapies that address the root causes of AO without compromising skin integrity or patient safety. Through this retrospective comparative research and detailed exploration, we aim to illuminate the complexities of this often-overlooked condition and highlight the urgent need for advancements in treatment to improve the quality of life of affected individuals.

## 2. Materials and Methods

### 2.1. Study Design and Population

Inclusion criteria included patients aged 18–60 years diagnosed with AO who had not responded to conservative treatments and were willing to undergo surgery. Exclusion criteria included patients with systemic diseases, history of keloid formation, or previous axillary surgery. Absolute indications included persistent and severe AO impacting the patient’s quality of life and social interactions. Contraindications included active infections in the axillary region, coagulopathy, and hypersensitivity to local anesthetics. This retrospective clinical study was conducted in a cohort of 30 patients (17 women and 13 men) who presented with AO and had an average age of 32.5 years. The participants were divided into two treatment groups: one undergoing laser-only surgery, and the other receiving combined laser treatment and liposuction surgery. The first group consisted of 18 patients (10 women and 8 men) who underwent laser treatment alone, whereas the second group consisted of 12 patients (7 women and 5 men) who underwent integrated liposuction following laser therapy.

### 2.2. Laser and Liposuction Procedures

A Nd:YAG laser (Accusculpt; Lutronic Co., Goyang, Republic of Korea) emitting a 1444 nm wavelength was used for laser surgery. The surgical procedure was performed using methods described in our previous paper [7]. The laser settings were standardized at a pulse rate of 40 Hz and energy output of 170 mJ. However, the total energy delivered and the duration of laser exposure were tailored to individual patient needs, with the total energy ranging from approximately 2000 J to 3400 J. The mean operating time for the first group, which underwent laser procedures alone, was approximately 45 min. In the integrated liposuction treatment group, liposuction was performed immediately following laser application to enhance the removal of the apocrine glands and fatty tissue. The mean operative time in the second group was 50 min.

**Figure 1:**
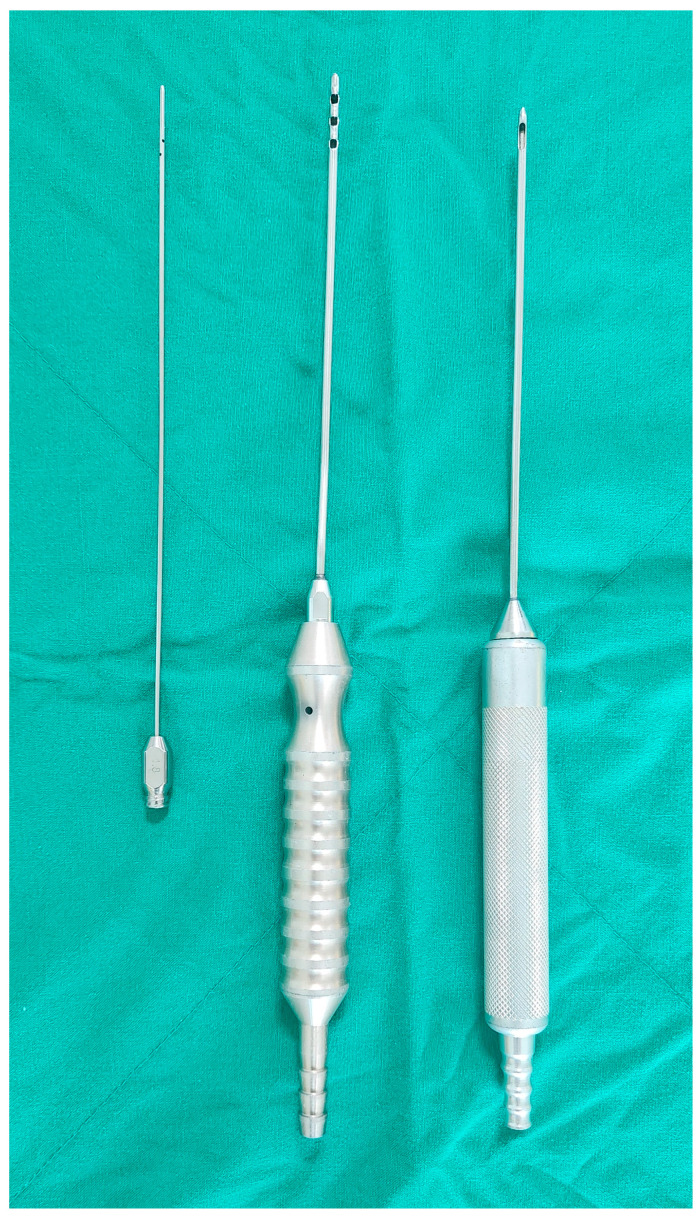
Cannulas used for liposuction to perform curettage of the apocrine gland.

**Figure 2:**
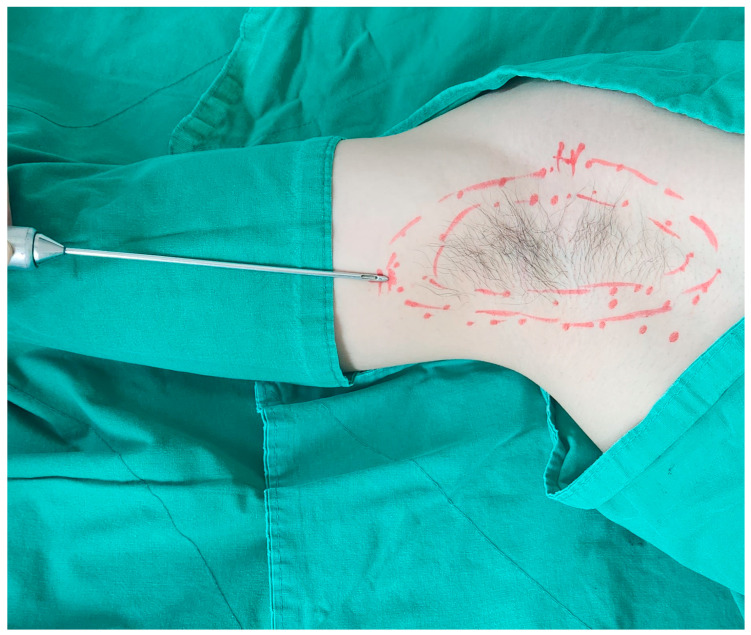
The design and method of liposuction of the surgical area.

**Figure 3:**
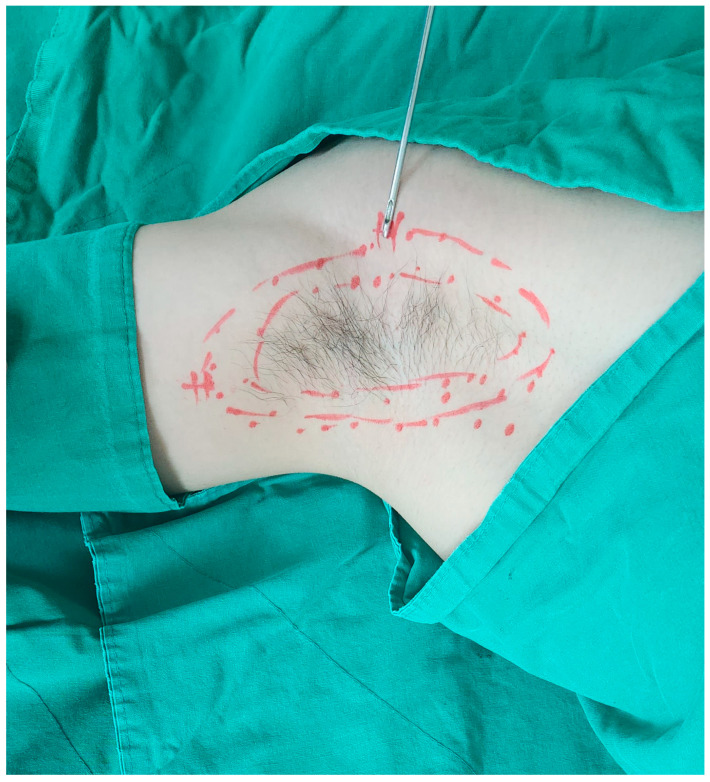
The design and method of liposuction of the surgical area.

Liposuction was performed using cannulas (Figure 1) with holes facing the skin to perform curettage of the apocrine glands. Two 5 mm incisions were made to ensure the overlap of the treated areas, minimizing the remaining apocrine glands. Approximately 5 mL of 2% lidocaine with 1:100,000 epinephrine was injected into the two incision sites, and 50 mL of tumescent solution (5 mL of 2% lidocaine + 0.2 mL of 1:1000 epinephrine + 50 mL of normal saline) was added for local anesthesia. The design and methods of liposuction are shown in Figure 2 and Figure 3, respectively. The liposuction procedure typically lasts approximately 10 min, at which point the underarm hair naturally begins to fall, indicating the end of surgery. Laser treatment before liposuction facilitates smooth cannula insertion, allowing for a more comfortable surgical procedure with minimal effort. A Penrose drain was inserted, and a figure-8 wrap was applied using 4-inch elastic bandages to prevent hematoma formation. The bandages were removed, and the drain was removed after 1–2 days. The sutures were removed after one week.

### 2.3. Outcome Measures

The outcomes assessed were changes in the degree of malodor (DOM), patient satisfaction, and the incidence of postoperative complications. DOM was measured using a gauze test performed in the outpatient clinic before treatment, at follow-up sessions after surgery, and at 7 days, 1 month, and 6 months postoperatively. The severity of DOM was classified on a scale from 0 (no odor) to 4 (strong odor detectable without direct contact; Table 1) [8].

### 2.4. Additional Diagnostic Tests

The sweat area test, conducted using the Minor Starch Iodine Test, was used to evaluate the efficacy of the post-treatment sweat reduction [9]. This involved applying a solution of iodine (1.5 g), castor oil (10 mL), and alcohol (100 mL) to the axillae, followed by dusting with cornstarch and exposure to an incandescent light bulb (500 W) at a distance of approximately 30 cm for 10 min, to facilitate the visualization of sweat production. The test was considered positive when the light-brown axillae turned dark purple owing to the formation of an iodine–starch complex. The area that tested positive in the patient’s axillary region was measured in cm^2^. Patient satisfaction was assessed using 4 grades: poor (0–25%), fair (26–50%), good (51–75%), and excellent (76–100%). Pain levels were quantitatively assessed using a visual analog scale (VAS) ranging from 0 (no pain) to 10 (maximum pain) and were recorded during surgery and on subsequent days post-surgery.

### 2.5. Safety Evaluation

To monitor the safety of the procedures, adverse reactions, such as burns, hematoma, infection, flap necrosis, partial hair loss, sensory loss, contracture, and thrombophlebitis, were systematically documented throughout the study.

### 2.6. Statistical Analysis

The data were analyzed using nonparametric methods owing to the ordinal nature of the DOM, satisfaction scores, and the potential non-normal distribution of pain scores. Continuous variables, such as DOM, satisfaction, and pain, were evaluated using the rank sum test, while categorical variables, including sweat area reduction and adverse effects, were analyzed using the chi-squared test. Since each participant had both right and left sides, all analyses were conducted to account for this clustered data structure. During this process, the Rao–Scott adjustment was applied to the chi-squared test. Statistical analyses were performed using the svytable and svyranktest functions of the survey package in R version 4.1.1.

## 3. Results

A total of 30 patients were enrolled in this comparative study. The patients enrolled in this study were divided into two groups. Group 1 comprised 18 patients, 44% of whom were male (n = 8) and 56% of whom were female (n = 10), with an average age of 32.9 years (SD = 13.9). Group 2 consisted of 12 patients, of which 42% were male (n = 5) and 58% were female (n = 7), with an average age of 30 years (SD = 15.39). Both groups were treated with a pulse rate of 40 Hz and an energy setting of 170 mJ. The mean total energy delivered was 2405.6 joules (SD = 417.9) on the right axillae and 2216.7 joules (SD = 355.2) on the left axillae for Group 1 and 2000 joules for both axillae in Group 2. The average procedure time was 45 min (SD = 11.6) for Group 1 and 50 min (SD = 4.4) for Group 2. The demographics and operative parameters of the 18 patients treated solely with laser therapy and the 12 patients who underwent liposuction following laser treatment are summarized in Table 2.

The findings from the comparative study assessing the efficacy of 1444 nm laser monotherapy versus integrated liposuction in treating axillary osmidrosis are summarized in Table 3.

**Figure 4:**
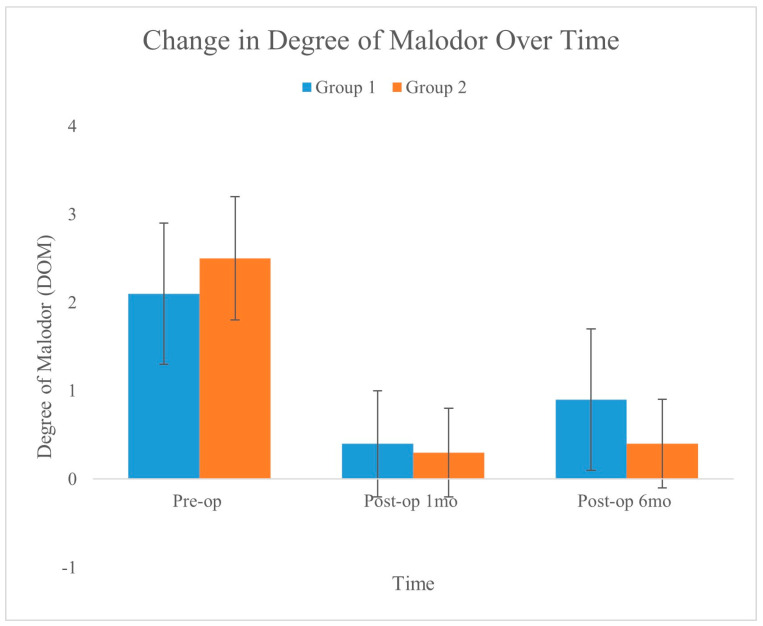
Changes in the degree of malodor were evaluated preoperatively and at 1 month and 6 months postoperatively. Although both groups showed a statistically significant reduction between preoperative and 6-month postoperative levels, the changes in Group 2 were greater than those in Group 1 (*p* = 0.002).

A significant reduction in DOM was observed in both the treatment groups. Group 2, which was subjected to integrated liposuction, showed a mean DOM reduction of −2.13 ± 0.19, which was significantly greater than the −1.09 ± 0.23 reduction observed in the laser monotherapy group (Group 1, *p* = 0.002). Figure 4 shows the changes in DOM over time for the two groups, as recorded preoperatively, one month postoperatively, and six months postoperatively. The graph shows a significant reduction in malodor in both groups, with the error bars indicating the standard deviation for each measurement. Compared to Group 1, Group 2 exhibited a slightly greater and more stable reduction.

**Figure 5:**
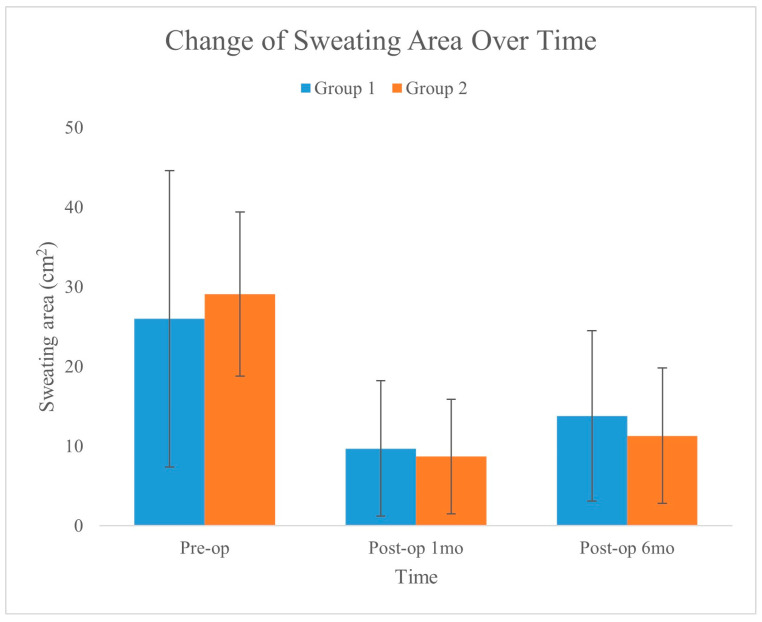
Changes in the sweating area using the minor starch–iodine test. Although the amount of sweating area in both groups decreased significantly between the preoperative and 6-month postoperative assessments, the changes in Group 2 were greater than those in Group 1 (*p* = 0.012).

There was a significant difference in sweat area reduction between the two groups, as determined by the sweat area test (*p* = 0.012). In Group 2, with integrated liposuction, all 24 patients experienced a decrease in sweating, whereas in Group 1, with laser monotherapy, only 19 of the 30 patients reported a decrease. Figure 5 shows the changes in sweat area over time for the two groups, as recorded preoperatively, one month postoperatively, and six months postoperatively. The graph illustrates a significant reduction in the sweating area for both groups, with the error bars showing the standard deviation for each measurement. This visualization highlights the effectiveness of the treatments in reducing the area of sweating over the analyzed periods.

**Figure 6:**
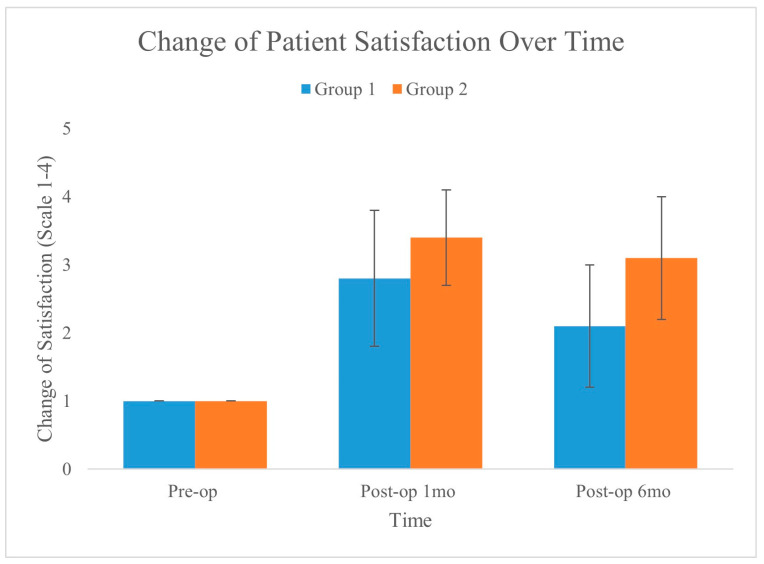
Changes in patient satisfaction were evaluated preoperatively and at 1 month and 6 months postoperatively. Although both groups showed a statistically significant increase between preoperative and 6-month postoperative levels, satisfaction in Group 2 was greater than that in Group 1 (*p* = 0.006).

Patient satisfaction also improved significantly in both groups, with integrated liposuction (Group 2) reporting a greater mean increase in satisfaction of 2.08 ± 0.25 than did the laser monotherapy (Group 1) 1.13 ± 0.21, with a *p*-value of 0.006. Figure 6 shows the changes in patient satisfaction over time for the two groups, as measured preoperatively, one month postoperatively, and six months postoperatively. The graph clearly shows an improvement in satisfaction from the preoperative baseline in both groups, with Group 2 exhibiting a greater increase and maintaining better satisfaction over time.

Both groups experienced a reduction in pain levels two days postoperatively. Although the difference was not statistically significant between the groups, the pain rating was higher in the liposuction group. The integrated liposuction group, Group 2, reported a mean pain reduction of −17.9 ± 2.36, compared to −9.82 ± 4.57 in the laser monotherapy group, Group 1 (*p* = 0.054).

There were significantly fewer burns in the integrated liposuction group, with only 4 (16.67%) patients experiencing this complication, compared to 22 (61.11%) in the laser monotherapy group (*p* = 0.025). There were no significant differences in swelling or contracture between the two groups. The percentage of patients without swelling was slightly higher in the integrated liposuction group (91.67%) than in the laser monotherapy group (88.89%); however, the difference was not statistically significant (*p* = 0.799). Similarly, contracture rates were comparable between the groups (*p* = 0.735).

This comparative study demonstrated that compared with laser monotherapy, integrated liposuction with 1444 nm laser therapy significantly improved the treatment outcomes of AO. The findings indicated that the combined approach achieved greater reductions in DOM, increased patient satisfaction, and effectively decreased sweat production, as evidenced by the significant results of the sweat tests. Statistically significant improvements were observed, with *p*-values ranging from 0.002 to 0.012 for primary outcome measures.

Moreover, compared to laser therapy alone, the integrated method significantly reduced the incidence of burns (*p* = 0.025), suggesting a safer profile for the combined treatment. However, no significant differences were found in swelling or contracture between the two groups, indicating that liposuction did not increase the risk of these complications.

Although pain was more pronounced in the integrated liposuction group, the difference was not statistically significant, suggesting that any increase in discomfort was offset by the enhanced effectiveness and safety of the combined approach.

Overall, the results indicate that compared with laser monotherapy, integrated liposuction combined with laser therapy significantly improves outcomes in terms of malodor reduction, patient satisfaction, and a lower rate of certain complications, such as burns. However, the differences in pain reduction and other complications, such as swelling and contracture, did not reach statistical significance.

## 4. Discussion

Axillary osmidrosis (AO), which is characterized by an offensive odor from the underarm region, is a multifaceted pathophysiological process. This condition results from the interaction between apocrine gland secretions, bacterial metabolism, genetic factors, and hormonal influences [10]. A deeper understanding of each component can aid the development of therapies and management strategies for this distressing condition.

Apocrine glands are specialized structures predominantly located in the axillary and genital areas [11]. Unlike eccrine glands, which primarily secrete water and salts, apocrine glands produce viscous, milky fluid rich in proteins, lipids, and steroids. This secretion is initially odorless but serves as a substrate for bacterial flora residing on the skin surface [12]. The onset of apocrine gland function coincides with puberty, which explains why AO is typically absent until adolescence [3].

The transformation of apocrine sweat into malodorous compounds is predominantly mediated by the skin-resident bacteria. The bacterial species involved in this process include *Corynebacterium*, *Staphylococcus*, and *Micrococcus* spp. [13]. These bacteria hydrolyze the lipids present in apocrine sweat into fatty acids and further degrade these compounds into smaller, volatile molecules, such as 3-methyl-2-hexenoic acid, which are primarily responsible for the characteristic odor associated with AO [2]. Recent studies have emphasized the role of microbial diversity in the intensity and nature of the odor produced, indicating that individual variations in the skin microbiota could explain the differences in scent severity observed among individuals [14].

One of the most significant determinants of AO is genetic variations, particularly in the ABCC11 gene [15]. The allele responsible for the production of dry earwax, commonly found in East Asian populations, is also associated with a significant reduction in axillary body odor. Approximately 80–95% of East Asians carry at least one copy of this nonfunctional allele, which correlates with the reduced secretion of odoriferous compounds by the apocrine glands. In contrast, populations of European, African, and other descent typically have a functional allele that leads to wet earwax and more active apocrine sweat production, thereby predisposing them to a more pronounced axillary odor. Furthermore, genetic predispositions affect not only the production of odor but also its perception, with variations in olfactory receptor genes influencing how odors are detected and processed by individuals [16].

Hormonal influences, especially androgens, significantly modulate the apocrine glands’ functions. Androgens increase the size and secretion in these glands. Fluctuations in hormone levels during different life stages, such as puberty, menstruation, and menopause, or conditions such as polycystic ovary syndrome (PCOS) can lead to changes in AO severity [17]. The hormonal sensitivity of the apocrine glands explains why symptoms can vary over time and why men typically experience more intense symptoms than women. Activation of apocrine glands at puberty marks the onset of potential AO, which continues to evolve throughout an individual’s lifetime. Furthermore, a decline in hormone levels associated with aging may lead to fewer symptoms in older adults. However, this is not universally observed, because individual variations in hormonal balance, health status, and lifestyle can affect the progression and severity of the condition.

Geographical factors, including climate and environmental conditions, significantly influence the epidemiology of AO. A warmer climate can exacerbate the perception of body odor owing to increased sweating, which provides more substrate for bacterial decomposition. Conversely, colder climates may reduce sweating but do not necessarily eliminate this condition, as odor-producing mechanisms remain active.

Cultural perceptions and hygiene practices play a critical role. In regions where body odor is socially stigmatized, there is a greater reporting of AO and a greater demand for medical interventions. Cultural norms influence not only the perception of the condition but also the willingness of individuals to seek treatment. Hygiene practices, such as the regular use of antiperspirants and bathing, can mitigate symptoms, but may also vary widely between different cultures and socioeconomic status.

AO is primarily recognized for its distressing clinical manifestation of persistent and strong body odor, which can persist even with rigorous hygiene practices [1]. This condition extends beyond mere physical symptoms and significantly affects psychological well-being and social interaction. The complexity of its clinical manifestations necessitates comprehensive understanding to facilitate effective management and therapeutic approaches.

The psychosocial impact of AO has not yet been overlooked. Stigma associated with bad body odors can lead to significant psychological distress, including low self-esteem, anxiety, social withdrawal, and depression [18]. Individuals may avoid social situations because of the fear of rejection or judgement, profoundly affecting their personal and professional relationships. This condition is frequently associated with a marked decrease in the quality of life, as patients navigate the challenges of managing a visibly unapparent but socially isolating condition.

Various surgical methods have been developed to treat AO, each of which features specific techniques, benefits, and limitations. Among surgical options, laser sweep ablation (LSA) is notable because of its minimal invasiveness [7]. It involves small incisions through which a laser fiber is inserted, and the laser energy is then delivered directly to the apocrine glands to destroy them and alleviate the subsequent malodor. This method is preferred because of its rapid recovery period.

Another technique, liposuction-assisted odor-corrective surgery, integrates liposuction with procedures to correct odor, addressing both excessive apocrine gland production and malodor [19,20]. Liposuction removes excess fat from the axillary region, which provides an environment for bacterial growth and contributes to the development of malodor. Following liposuction, additional procedures, such as gland excision or laser therapy, may be performed to address sweat gland activity and odor production.

Surgical excision of the apocrine glands in the axillary region is another approach for managing AO [21]. This procedure involves removing the apocrine glands either through traditional surgical excision or minimally invasive techniques, such as endoscopic or subcutaneous excision. By removing the apocrine glands responsible for odor production, this method aims to permanently reduce the malodor. However, it requires a long recovery period and severe scarring, and the recurrence rate of osmidrosis is not significantly lower than that of other methods. Therefore, we do not recommend this approach.

For those seeking nonsurgical options, botulinum toxin injections can be used to temporarily block sweat production in the axillae [22]. The principle is to treat axillary bromhidrosis without traditional surgery, which can be a good method for reducing osmidrosis, requiring a short-term recovery period. The literature review indicates that botulinum toxin provides temporary relief from AO symptoms but may require repeated treatments [6]. Botulinum toxin is injected into the skin of the axillary region, where it inhibits the release of acetylcholine, a neurotransmitter involved in sweat and apocrine gland activation. This results in decreased gland production and temporary malodor relief.

Microwave thermolysis is a relatively new AO treatment. This approach involves the application of microwave energy to the axillary skin, which selectively targets and destroys apocrine glands. Compared with traditional surgical techniques, a microwave-based device (miraDry system; MiramarLabs, Santa Clara, CA, USA) offers the advantages of being minimally invasive and providing good results with fewer side effects [23,24].

Among innovations in treatment modalities, radiofrequency (RF) technology has emerged as a significant method. Fractional microneedle radiofrequency (FMR) devices specifically deliver energy to the deep dermal layers through insulated microneedles, sparing the epidermis from damage [25]. These devices were initially proven to be efficacious in the management of wrinkles, acne scars, and enlarged pores. Oh et al. achieved promising outcomes in the treatment of primary axillary hyperhidrosis (PAH) using fractional microneedle radiofrequency (FMR) treatment (Infini^TM^; Lutronic, Goyang, Republic of Korea) [26]. Although PAH and osmidrosis differ in their clinical presentation, they frequently coexist. Consequently, it appears viable to explore the application of the FMR technology in the treatment of osmidrosis.

Each surgical method for AO has its own benefits, risks, and outcomes. The choice of technique depends on factors such as the severity of the condition, patient preferences, and expertise of the surgeon. It is essential that patients discuss their treatment options with a qualified healthcare provider to determine the most suitable approach for their individual needs.

Compared with these methods, standalone laser treatment is typically less invasive, resulting in less skin damage. Patients treated with lasers generally experience a shorter recovery period and less postoperative pain. They also tend to have a lower likelihood of complications, making them safer. However, laser treatment alone may not provide a permanent solution, and there is a possibility of recurrence. In the laser treatment alone group, there were patients who did not respond, indicating a higher recurrence rate with laser monotherapy. The effectiveness of the treatment can vary significantly among individuals owing to differences in the skin tissue response. This recurrence is considered to be the greatest disadvantage of laser treatment alone.

By contrast, combining laser surgery with liposuction often provides more durable and comprehensive outcomes. Liposuction removes excess tissue, whereas laser treatment diminishes the activity of apocrine glands, yielding lasting results. Liposuction may reduce the likelihood of malodor recurrence by extracting fat tissue surrounding the apocrine glands. Liposuction is a surgical procedure that carries the risks of complications and skin damage. Additionally, when both procedures are performed concurrently, the recovery period may be prolonged, and surgical expenses can increase.

Selecting an appropriate treatment for AO requires careful consideration of individual patient factors, such as symptom severity, lifestyle, and personal preferences, as well as the surgeon’s expertise and available technology. It is crucial for patients to understand the potential outcomes, risks, and benefits of each option in order to make well-informed decisions. The cost is a critical factor in treatment selection. While the initial cost of the combined procedure might be higher due to the involvement of both laser and liposuction equipment, the improved patient satisfaction, reduced need for repeat procedures, and lower complication rates can lead to overall cost savings in the long term. Additionally, the shorter recovery period associated with our method can reduce indirect costs, such as time off work and associated healthcare expenses.

This study has several limitations, such as the small sample size, relatively short observation period, insufficient liposuction standardization, and the inability to assess malodor in ordinary days. The primary reason for the limited sample number was the strict inclusion criteria. We conducted an analysis of surgical outcomes among patients at university hospitals, where the number of surgeries for axillary hyperhidrosis is limited. We acknowledge that the small sample size may restrict generalizability and suggest that concurrent liposuction and laser therapy could be a promising alternative for refractory axillary hyperhidrosis. Future studies could benefit from a multicenter approach to increase the sample size and strengthen the findings. We agree that a six-month follow-up may not be sufficient to evaluate long-term outcomes and complications adequately. Challenges in maintaining patient participation beyond six months influenced our study’s design, initially set for a six-month duration. We acknowledge the need for extended follow-up (one to two years) in future research to assess treatment durability and late-onset complications. There is a pressing need for ongoing research to enhance the existing treatments and develop new technologies. Recent progress in genetics and microbiome studies promises more targeted approaches that could address the root causes of excessive sweating and malodor more effectively. Collaborative efforts among dermatologists, plastic surgeons, and researchers are vital for advancing treatment protocols and improving the outcomes of patients with AO.

## 5. Conclusions

Integrated liposuction with laser therapy offers a more effective and potentially safer alternative for treating axillary osmidrosis (AO), providing long-lasting relief from symptoms and a greater degree of patient satisfaction. Future research should focus on optimizing these techniques to further improve the safety and efficacy of treatment and explore the potential for even less invasive options that could provide similar benefits.

## Figures and Tables

**Table 1:** Malodor grading system. Reproduced with permission from [7].

Grade	Degree of Malodor
0	Gauze is not malodorous
1	Gauze is slightly malodorous (when carefully sniffing the gauze)
2	Gauze is malodorous (with the gauze immediately in front of the face)
3	Gauze is strongly malodorous (can be smelt when holding the gauze away from the face)
4	Axillae are malodorous and can be smelled even before the gauze is held under the patient’s arm

The degree of malodor was classified into 5 grades, obtained using gauze held under the patient’s arm for 1 min.

**Table 2:** Patient demographics and operative parameters.

	Patients (N)	Male, n (%)	Female, n (%)	Age (Year) (Mean ± SD)	Pulse (Hz)	Energy (mJ)	Total Energy Right (J) (Mean ± SD)	Total Energy Left (J) (Mean ± SD)	Time (min) (Mean ± SD)
Group 1	18	8 (44)	10 (56)	32.9 ± 13.9	40	170	2405.6 ± 417.9	2216.7 ± 355.2	45 ± 11.6
Group 2	12	5 (42)	7 (58)	30 ± 15.39	40	170	2000	2000	50 ± 4.4
Total	30	13 (43)	17 (57)	31.7 ± 14.2	40	170	2189.8 ± 342.2	2227.7 ± 368.1	47 ± 9.2

**Table 3:** Summary of the results.

Outcome		Laser Monotherapy	Integrated Liposuction	*p*-Value
Degree of malodor (Changes, mean ± SE)		−1.09 ± 0.23	−2.13 ± 0.19	0.002 *
Degree of malodor (Changes, mean ± SE)		1.13 ± 0.21	2.08 ± 0.25	0.006 *
Pain level (Changes, mean ± SE)		−9.82 ± 4.57	−17.9 ± 2.36	0.0540
Sweat test				0.012 *
	non-decrease (n)	11	0	
	decrease (n)	19	24	
Complications				
Burn				0.025 *
	No (n (%))	14 (38.9)	20 (83.3)	
	Yes (n (%))	22 (61.1)	4 (16.7)	
Swelling				0.7990
	No (n (%))	32 (88.9)	22 (91.7)	
	Yes (n (%))	4 (11.1)	2 (8.3)	
Contracture				0.7350
	No (n (%))	34 (94.4)	22 (91.7)	
	Yes (n (%))	2 (5.6)	2 (8.3)	

* *p* < 0.05 is statistically significant. SE: standard error.

## Data Availability

The data underlying this article will be shared upon reasonable request to the corresponding author.
